# Who Has Access to Livestock Vaccines? Using the Social-Ecological Model and Intersectionality Frameworks to Identify the Social Barriers to Peste des Petits Ruminants Vaccines in Karamoja, Uganda

**DOI:** 10.3389/fvets.2022.831752

**Published:** 2022-02-28

**Authors:** Daniel Acosta, Nargiza Ludgate, Sarah L. McKune, Sandra Russo

**Affiliations:** ^1^Department of Environmental and Global Health, College of Public Health and Health Professions, University of Florida, Gainesville, FL, United States; ^2^International Center, University of Florida, Gainesville, FL, United States

**Keywords:** gender, intersectionality, livestock vaccine value chain, peste des petits ruminants, Karamoja, Uganda, socio-ecological system

## Abstract

Access to veterinary services is important in Karamoja, northeastern part of Uganda, as livestock is a primary source of livelihood. Gender is often overlooked in animal health programs, let alone intersectionality. However, given the socio-cultural intricacies of Karamoja, ignoring these factors may hinder animal vaccination practices, limiting the success of programs designed to control and prevent animal diseases, such as peste des petits ruminants (PPR). The study used qualitative research methods, including focus group discussions, individual interviews, and key informant interviews in a participatory research approach to investigate the constraints faced by livestock keepers when accessing vaccines. The study was carried out in Abim, Amudat, Kotido, and Moroto, four districts in the Karamoja Subregion of Uganda. A modified version of the socio-ecological model (SEM) blended with an intersectional approach were used as frameworks to analyze underlying individual, social and structural determinants of vaccine access with intersecting factors of social inequalities. The results show there are seven intersecting factors that influence access to vaccination the most. These are: gender, ethnicity, geographic location, age, physical ability, marital status, and access to education. The impact of these intersections across the different levels of the SEM highlight that there are vast inequalities within the current system. Access to vaccines and information about animal health was most limited among women, widows, the elderly, the disabled, geographically isolated, and those with unfavorable knowledge, attitudes, and practices about vaccination. Cultural norms of communities were also important factors determining access to PPR vaccines. Norms that burden women with household chores and beliefs that women cannot manage livestock, combined with gender-based violence, leaves them unable to participate in and benefit from the livestock vaccine value chain. Trainings and sensitization on gendered intersectional approaches for those involved in the distribution and delivery of vaccines are necessary to avoid exacerbating existing inequalities in Karamoja.

## Introduction

Livestock are one of the primary sources of livelihood for many Ugandans, with 60% of smallholder farmers raising livestock for sale and home consumption (1). Livestock alone contributed 4% of the gross domestic product (GDP) in 2018 (2). The largest livestock herd in Uganda is located in the Karamoja subregion, where livestock also hold important cultural significance (3). Karamoja is frequently described as the poorest and most socio-politically marginalized region of Uganda, with most of its population relying on livestock as a main source of livelihood (4). There are many constraints to livestock development in Karamoja, including limited access to veterinary services (3), lack of infrastructure, human development (5), and an overall historical neglect of the subregion (6). Given Karamoja's geographic proximity to bordering Kenya and South Sudan and the agropastoral and pastoral practices of its population, improving access and uptake of veterinary services, such as vaccines, could combat the spread of livestock diseases in East Africa. Peste des petits ruminants (PPR) is a viral disease endemic across East Africa that affects both sheep and goats. PPR devastates livestock communities and causes economic losses across the world estimated at around 1.5–2 billion US Dollars a year, which is why it is targeted for eradication by 2030 (7). Given how women in pastoralist settings often have access to small ruminants, with little to no access to large ruminant production and supply chain, they can be disproportionately affected by small ruminants diseases, such as PPR (8–10). The Karamoja subregion alone has two hotspots of virus transmission, one located in two adjacent sub counties of Kotido and Kaabong districts, and another in Amudat district, on the Uganda-Kenya border (11), underscoring the importance of controlling the disease in this area. Livestock vaccination campaigns in Karamoja are mostly, if not entirely, undertaken by the government, and in coordination with the non-governmental organizations (NGOs) (12); however, funding for livestock vaccines is insufficient, information about vaccines is scarce among smallholders, and cold chain capacity is inadequate (5). The private sector is essentially non-existent and does not have the capacity nor regulatory environment to source and maintain vaccines for sale. Overall, access to veterinary services is challenging, with insufficient and inconsistent supplies further exacerbated by inadequate veterinary delivery infrastructure and human capital (5, 13).

**Figure 1:**
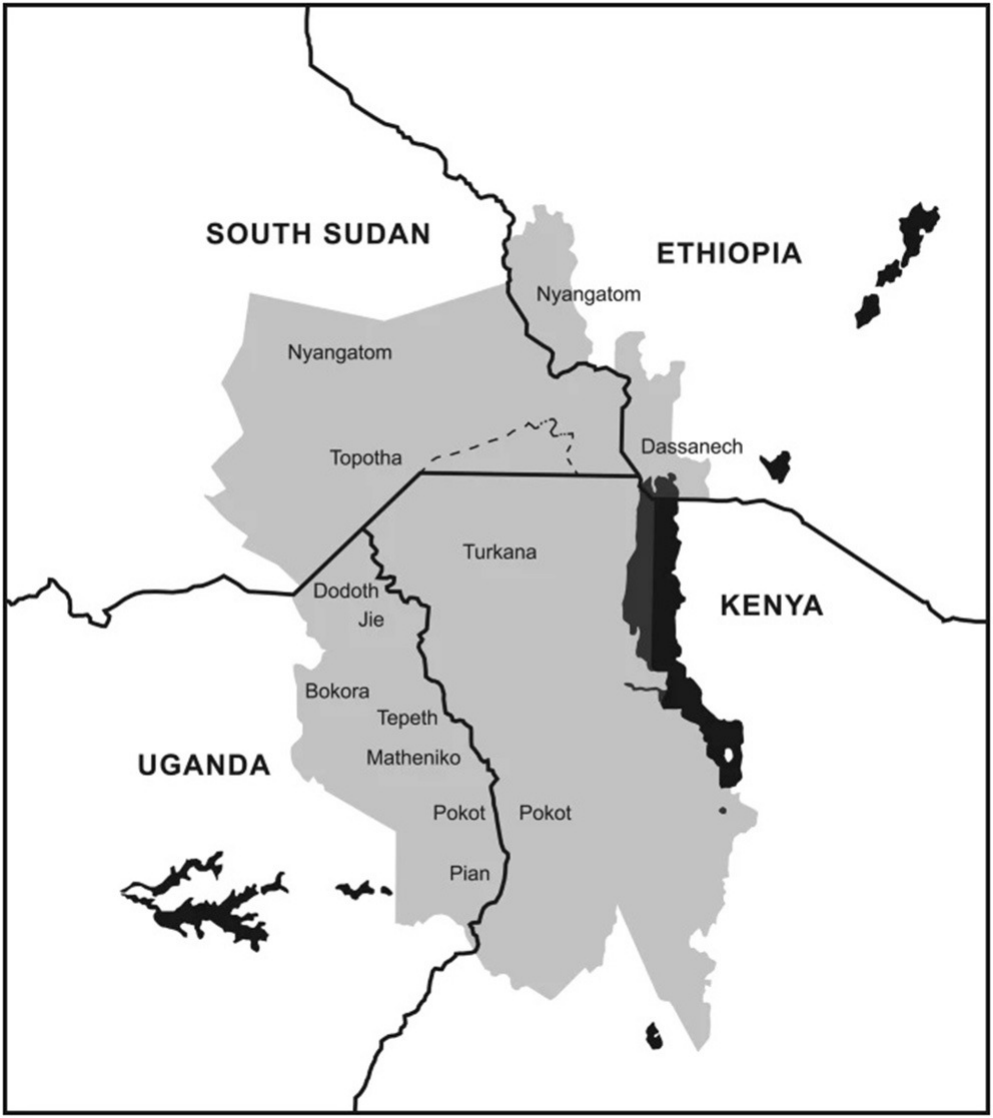
Map of the Karamoja cluster obtained from (20).

Moreover, this semi-arid region experiences chronic food insecurity, has over 60% of the population living below the poverty line, a fertility rate of eight children per woman, and low economic productivity (<1% of Uganda's total GDP) (14–16). The region also has very porous borders with neighboring countries of Kenya and South Sudan, especially in the dry season when pastoralists migrate in search of pastures and water. Additionally, Karamoja has a violent history marked with armed inter and intra communal conflicts (17). Despite the disarmament of communities a decade ago, which significantly reduced systematic cattle raiding, violence in other forms such as sporadic cattle raiding, theft, sexual assault, communal violence, clan-related warfare, and gender-based violence are still common (15, 18, 19). Up to 53% of women have experienced physical violence, with 49% of women and 43% of men indicating it is justifiable for a man to beat his wife (15). Figure 1, from (20), shows a map of the Karamoja Cluster, which is the geographical area where Karamoja is located, and an approximate geographic location of the largest ethnic groups in the region. The relationship between the different ethnic groups such as the Jie, Matheniko, Ethur, Pokot, and the Tepeth has been marked by violence and death in the past few decades, due mainly to the use of automatic weapons during cattle raids, often referred as “AK-47 raids” (21). Violence between the different ethnic groups in Karamoja has a complex history and was not limited to the Ugandan border, as conflicts and raids also included pastoralist communities from Kenya and South Sudan. The escalation of conflict led to attacks beyond cattle raiding, with schools in the Turkana region of Kenya and the Karamoja region of Uganda suffering the consequences and resulting in the deaths of many children (21, 22). Finally, despite inter-ethnic violent relations, Karamoja is considered an ethnically diverse region where different ethnicities share the common interest of livestock rearing and adjusted their lifestyles and culture around livestock. Therefore, the importance of considering ethnicity as it intersects with gender and other social markers in activities related to livestock is especially important in the context of Karamoja, as there is an intricate history of conflict and culture surrounding livestock and ethnicity in the region.

As the Government of Uganda works to mainstream gender into agricultural production, the Karamoja subregion continues to face the greatest challenges (23). Livestock interventions often ignore how gender dynamics influence the uptake of technologies (24), and even when gender is considered as the variable of interest for such interventions, the intersection of gender with other social markers (such as ethnicity, class, religion, etc.) is often overlooked (5, 25). There is growing consensus about the importance of *intersectionality* in agriculture, as it plays a crucial role in understanding women's empowerment (26). Intersectionality is often defined as a framework that analyzes how different social characteristics, such as race, gender, ethnicity, class, or age overlap and create a system of advantages and disadvantages (27–30). Intersectionality is a relatively recent framework that has gained traction in the social sciences and public health as a critical tool both to analyze data and to design effective interventions by understanding and addressing the experience of populations defined by layered identities (e.g., not just men vs. women, but poor and minority women) and how that intersectional identity drives their interaction in different systems (29, 31). In the context of livestock vaccinations, the framework of intersectionality has been proposed as a tool to facilitate an understanding of the dynamic relationship of inequality and marginalization, by illustrating which livestock keeper or group faces greater and differential barriers to accessing vaccines (25, 32). Given Karamoja's diverse ethnicities and the turbulent history of ethnic conflict, the intersection of gender and ethnicity, along with other social markers, becomes particularly important when it comes to livestock, as livestock are deeply embedded in cultures, norms, and livelihoods of various ethnicities residing in the region.

Intersectionality sheds light on the intricate, nuanced experience of women in Karamoja when comparing ethnic groups. All groups practice agro-pastoralism and raising livestock in kraals (mobile camps). Despite similarities, their practices also have differences in livestock rearing and marketing, often surrounded by and understood within social and cultural norms, particularly those norms that define gender roles and engagement with livestock across the region (33). Though there are many different practices and beliefs surrounding livestock in the different ethnic groups of Karamoja, there is one constant: men control livestock, especially cattle. Women are usually in charge of growing sorghum and maize (34, 35). Some women, especially among the Jie, Matheniko, and the Tepeth, can be involved in certain livestock activities (watering, checking for disease, caring for the sick animals, and cleaning) but rarely sell livestock (36). Where women own livestock or have some level of decision-making power, it is mainly small ruminants and poultry, which is why this study focuses on women's ability to access PPR vaccines. While most women do not have decision-making power over the sale and management of proceeds from sale, small ruminants remain important sources of cash and protein for some women and children, especially when their husbands are far away grazing livestock (11). Despite the unique, intricate, and diverse social norms surrounding livestock in Karamoja, animal health (including vaccination) programs rarely consider ethnic differences of livestock keepers, thus miss opportunities to optimize success of their interventions (5).

Given the complex and intertwined socio-cultural beliefs, gender norms, socio-demographic characteristics, geographic factors, and livestock management in Karamoja, qualitative research methods are well-suited to understand the nuances surrounding the norms and practices of livestock vaccination. This study uses a modified version of the Social Ecological Model (SEM) adapted from Tully et al. and Sallis et al. (37, 38) and an intersectional analysis to map the livestock vaccine value chain (LVVC) to better understand how gender, ethnicity, and other social markers intersect to exacerbate inequality and marginalization in livestock keepers' access to PPR vaccines. The SEM was conceptualized in the 1970s by Bronfenbrenner, as a framework to understand the effects of different factors (personal and environmental) in human development at different levels (39–41). The levels originally proposed by Bronfenbrenner were the microsystem, the mesosystem, the exosystem, and the macrosystem. Each of those levels surrounded the individual and became progressively broader; for instance, the microsystem comprises an individual's surroundings (e.g., peers, family), while the macrosystem comprises societal influences (e.g., policies, regulation). Since then, the use of the SEM has risen—particularly in public health—and new versions of the SEM with different levels than those originally proposed have become increasingly common, with organizations like the U.S. Center for Disease Control and Prevention, and the World Health Organization adopting their own version of the SEM (42, 43). The SEM has been used to develop effective behavior change interventions of all sorts, including increasing human vaccine uptake (44, 45). There is a growing body of literature that calls for the adaptation of social-ecological systems and models toward a One Health approach, inclusive of veterinary medicine (46, 47). A few studies have used both SEM and intersectionality to understand socially complex situations (48–50). The use of both approaches allows stakeholders to understand how the intersection of different social markers could exacerbate or alleviate barriers at different levels in complex systems, but the integrated use of these approaches remains low and is, to our knowledge, inexistent in understanding livestock vaccines.

“*Who has access to livestock vaccines?*” is an important question to ask and answer before planning and undertaking any vaccination effort. Systematically leaving certain groups marginalized, disengaged, or neglected from vaccination campaigns risks exacerbating existing inequalities and hindering control, elimination, or eradication efforts. Given the gendered dynamics of livestock production, with women having more input into and potential to benefit from goats and sheep, understanding access to vaccines for small ruminants through an intersectional lens becomes critical. Efforts to eradicate PPR further focus the scope of our analysis to the PPR vaccine specifically. With Karamoja being such a remote and ethnically diverse setting, a gender analysis is insufficient to developing appropriate strategies to increase vaccination; any intervention strategy to optimize vaccination uptake, a requisite step toward eradication, must include an analysis of different intersectional factors in the population. A well-designed study that integrates the SEM and an intersectional approach can illuminate how layers of influence, from policy down to individual level barriers, drive what appears to be an individual decision—to vaccinate livestock—and may identify points of intervention for improved vaccination strategy. This manuscript utilizes an intersectional approach as a framework to identify the barriers faced by livestock keepers to obtain PPR vaccination. The SEM is used to position those barriers at different levels and evaluate their overlapping effects.

## Materials and Methods

The qualitative research design for mapping the LVVC drew on expertise from gender studies, economics, animal sciences, veterinary medicine, and extension to evaluate the intersection of gender with other intersectional markers and their influence on women's engagement in the LVVC. Data collection mostly took place in four districts of the Karamoja subregion of Uganda (Abim, Amudat, Kotido, and Moroto). Data were also collected in Kampala and Entebbe with national level stakeholders and government officials. The selection of the districts was determined by the presence of PPR hotspots and the significant ethnic differences among the districts (11, 51). Data were collected in two phases, the first in November-December 2019 and the second in January-February 2020. Preliminary results from the first phase of data collection were shared with stakeholders in early January 2020, which led to minor adjustments in the data collection strategies prior to the second round. The study was cross-sectional involving participatory qualitative methods of Focus Group Discussions (FGDs), Individual Interviews (II), and Key Informant Interviews (KIIs). The local field team (interviewers and note takers) were trained by researchers from the University of Florida in the use of research instruments and participatory methodologies, as there are many dialects and languages that are only spoken in the Karamoja subregion. The data collection team was divided into two groups, those collecting data in the northern area (Abim, Kotido, and Moroto) and those collecting data in the southern area (Amudat). The team collecting data in the north was composed by two women from Abim that resided in Moroto and one man from Kotido, all whom were either native or fluent speakers of the languages and dialects of the areas. Enumerators in the southern area were two Pokot women from Amudat, with associated language skills. Ethical approvals for the study were obtained from the University of Florida's Institutional Review Board (#IRB201901128) and the Higher Degrees Research Committee at the Makerere University (# SBLS/HDRC/19/007b). The study also obtained a research clearance from the Uganda National Council for Science and Technology (#A608). Written informed consent was obtained from participants, and those who were not able to sign provided a thumb print to consent. Participants did not receive monetary compensation for their participation, only refreshments and transportation reimbursement (when applicable, in accordance with ethical protocols).

A total of 22 KIIs, 20 IIs, and 40 FGDs were conducted using purposive sampling distributed as shown in Table 1. The KIIs were conducted at different administrative levels: seven at the national level, five at the regional level, six at the district level, and four at the community level. Based on the past experience conducting research in Uganda and semi-structured interview protocol, the KIIs were not recorded and relied on detailed notetaking by the research team. KIIs at the national and regional level were done in English; some local level KIIs required a local translator and note taker. Notes from the KIIs were also included in the analysis to explain and expand on the perceptions and barriers to vaccination by key actors in the value chain. The IIs aimed to understand individual barriers to vaccination faced by downstream value chain actors (i.e., livestock keepers as users, and community animal health workers as community-based vaccinators). The FGDs were conducted with livestock keepers and with Community Animal Health Workers (CAHWs), separately. The FGDs with livestock keepers sought to understand participants' experiences with small ruminant rearing, access to and information about vaccines, experiences of past vaccinations, advantages and disadvantages of livestock vaccinations, and barriers faced in accessing vaccines. The FGDs with CAHWs investigated the day-to-day responsibilities of the community-based livestock service providers, supply and demand drivers among livestock population, barriers faced by CAHWs and livestock keepers in accessing vaccines, and their experience in working with communities and district veterinary service providers during vaccination campaigns. A detailed account of the number of FGDs by district is shown in Table 2. FGDs were disaggregated by gender (men and women), occupation (CAHW and livestock keepers), and ethnicity (largest ethnic groups in the region). The number of participants in each FGD was generally 6–10, with few exceptions where the number of participants was 11 or 12. The number of participants per FGD remained within range of what the research team considered appropriate, which also coincides with recommendations from the literature (52, 53). FGDs and IIs were recorded, transcribed, and translated into English. Data from the transcripts were de-identified in accordance with ethical guidelines. These transcripts were examined using Exploratory Thematic Analysis, a methodology for qualitative data analysis where themes and patterns are systematically identified across a data set (54–56). The analysis process was iterative, with a pilot round of coding to identify emerging themes, which included gender constraints to accessing information about livestock, vaccine distribution issues, myths surrounding vaccines, issues with CAHWs, and others. In later iterations of analysis, these themes were corroborated, expanded upon, or refined, as certain themes across gender and different ethnic groups started to become recurrent. A modified version of the SEM (37, 38), which incorporated an intersectional approach to explore the intersecting dimensions (50), was used to illustrate the factors that could be barriers to PPR vaccination practices in Karamoja. This version of the SEM has four levels: Individual, Social Environment, Physical Environment, and Policy Levels. The Individual Level contains those factors that could be considered unique to each livestock keeper and CAHW in a given environment, but excludes social characteristics given that these are analyzed as intersecting elements. The Social Environment Level are those social characteristics that are unique and shared among a group of individuals, such as the residents of a village, or members of a cooperative. The Physical Environment Level contains factors that could physically impact access to participating in the LVVC. The Policy Level encompasses broader enabling environment characteristics, such as those dictated by national governing bodies and international organizations.

**Table 1:** Data collection instruments.

**Instrument**	**District**	**Actors involved**	**Total**
FGDs*	Abim, Amudat, Kotido, Moroto	CAHWs, Livestock Keepers	40
KII	Kampala and Entebbe (cities) Abim, Amudat, Kotido, Moroto	NGO personnel at national and regional level, veterinary suppliers (private sector), government staff [national and district level, e.g., district veterinary officers, staff at the Ministry of Agriculture, Animal Industries, and Fisheries, university researchers, community and kraal leaders (community level)].	22
II	Abim, Amudat, Kotido, Moroto	CAHWs, Livestock Keepers	20

*FGDs were mostly sex-disaggregated, but in some instances, due to lack of women CAHWs, they were mixed.

**Table 2:** Detailed distribution of FGDs held in each district.

**District**	**Ethnic group**	**Actors involved**	**Number of interviews/types of actor/Ethnic Group**	**Total**
Abim	Ethur	CAHWs, Livestock Keepers	CAHWs (2FGDs, one with men and one with women) Livestock keepers (4FGDs, two with men and two with women)	6 FGDs
Amudat	Pokot	CAHWs, Livestock Keepers, Agro veterinary store owners	CAHWs (2FGDs, one with men, one with women) Livestock Keepers (12FGDs, six with men, six with women)	14 FGDs
Kotido	Jie	CAHWs, Livestock Keepers	CAHWs (3FGDs, one with men*, one with women, one mixed genders*) Livestock Keepers (6FGDs, three with men and three with women)	9 FGDs
Moroto	Matheniko	CAHWs, Livestock Keepers	CAHWs (4FGDs, two with men, one with women, one mixed genders*) Livestock Keepers (4FGDs, one with men, two with women, one mixed genders*)	8 FGDs
	Tepeth	Livestock Keepers	Livestock Keepers (3FGDs, two with men**, one with women)**	3 FGDs

*Indicates when FGDs had both men and women. **One of the FGDs had a few participants that identified as Matheniko.

## Results

**Figure 2:**
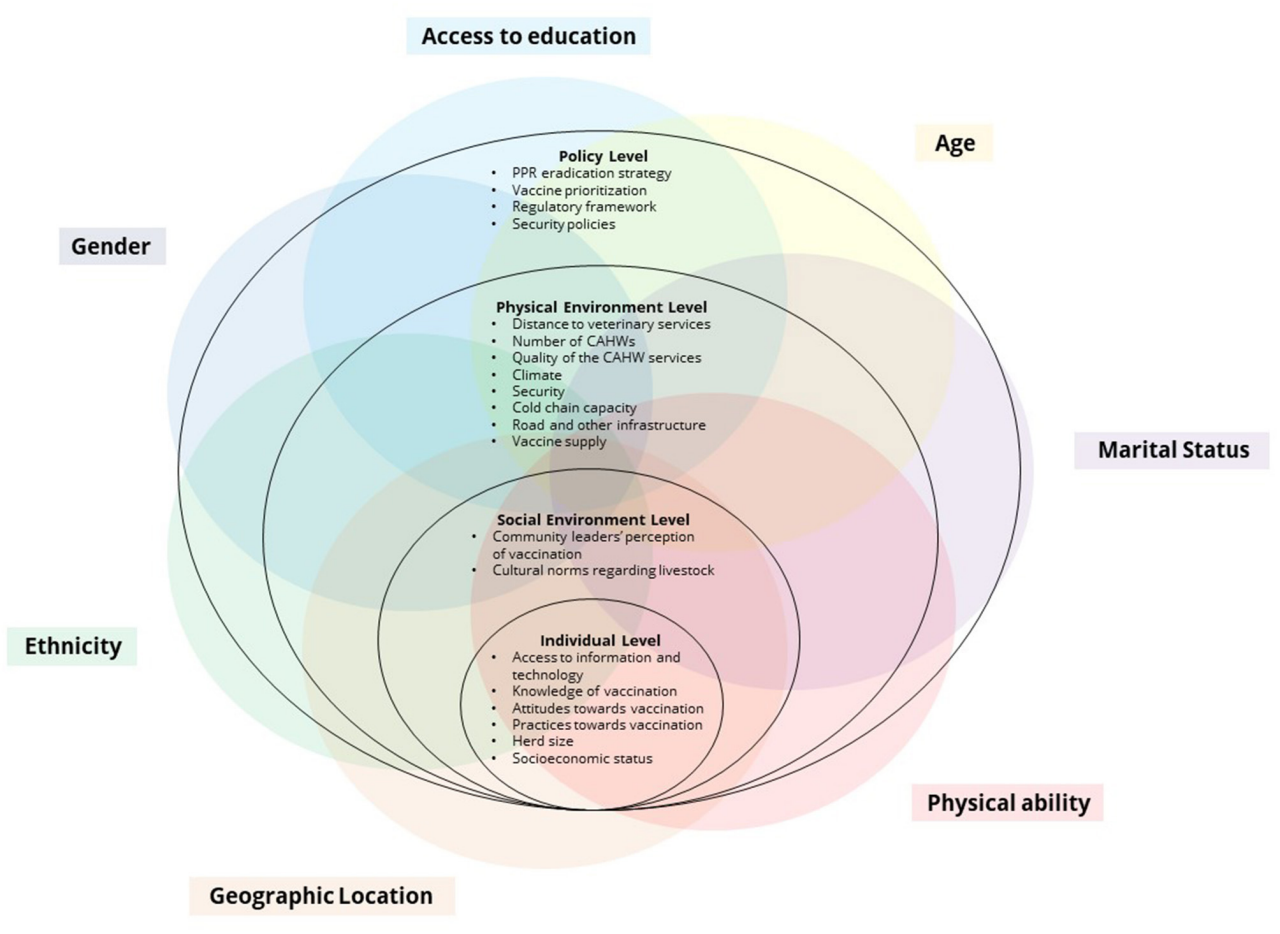
SEM and Intersectionality frameworks for PPR vaccinations in Karamoja. The SEM was adapted from (37), and the combination of the SEM with the intersectional framework was adapted from (50).

Figure 2 illustrates the factors livestock keepers interact with when it comes to accessing PPR vaccines at each socio-ecological level and the intersections across each [the framework was adapted from (50)]. The intersectional lens illustrates how, even though some barriers are ubiquitous among livestock keepers (e.g., those at the Policy Level), the intersection of different social markers influences how these barriers are experienced. The seven intersections identified are: gender, access to education, age, marital status, physical ability, geographic location, and ethnicity. Access to education references both the ability to attend school, and the ability to attend trainings (e.g., trainings in agricultural production). Geographic location is characterized as the space where an individual is settled and how accessible vaccines and other animal health services are in this area. Sometimes accessibility to vaccines is directly related to distance to major urban areas and infrastructure, but this is not always the case, as there are also power dynamics involved in which some individuals or groups might receive preference (e.g., livestock keepers with larger herds).

### Individual Level

At the Individual Level, we identified access to information; knowledge, attitudes, and practices toward vaccination; herd size; and socioeconomic status (SES) as having a direct effect on whether a livestock keeper will be able to access PPR vaccines. The impact of these elements fluctuates depending on the intersecting factors identified above. For example, practices and beliefs vary greatly depending on the ethnic group, geographic location, education level, and gender. Attitudes toward vaccination had some variation by ethnic group. The Jie livestock keepers had the least concerns about vaccine safety and, overall, showed greater willingness to vaccinate compared to livestock keepers from the Ethur ethnic group, some of whom expressed reluctancy to vaccinating their livestock. Socio-economic status also impacted access to vaccines, as occasionally vaccines have a service fee attached to them, therefore, people with less economic resources (lower SES) faced greater constraints. Similarly, the size of the herd was also identified as a deciding factor when it came to access to vaccines or willingness to vaccinate (it should be noted that we didn't probe willingness to vaccinate in the study). Veterinary services were perceived to prioritize larger herds, which are usually owned by men, or those areas where livestock keepers demanded vaccines (the Jie livestock keepers).

Access to information about vaccines was gendered and varied by marital status, with more information about vaccination programs reaching men than women, and among women, less information reaching widows [similar phenomenon was observed in Senegal, see (25)]. In several focus groups, women livestock keepers mentioned their desire to see more women CAHWs, as they felt this would make it easier for them to access information (See Table 3). One of the key processes where women are most affected by the lack of access to information is during mobilization for vaccination activities. Regional and district level mobilization are done through sub-county animal husbandry staff, local leaders, and focal points within the communities. As men control most of the livestock and information spaces (e.g., various communication channels and mobile phones), it is they who receive information on vaccines. Because most livestock are in control of men, and men are usually in decision-making positions, information is mostly passed to them. This is done under the assumption that men are the sole decision makers of livestock production. A community leader from Kotido, who has often served as mobilizer for livestock vaccines said “Culturally, women are not allowed to own animals including goats and sheep. When women buy animals, that animal belongs to her husband. (…)The animal of women is the granary; they have to look after sorghum, millet, groundnuts in the granary.” However, one of our significant findings is that even though most women might not have the decision-making agency over livestock, some are highly involved in the care and health of animals, especially small ruminants and poultry. Another assumption, which was not validated, may be that men will pass on information to their wives or other women in their households, including widows. Marital status then becomes an important intersecting factor when it comes to accessing highly gendered information about vaccines space. Women who are widows can own and control livestock in most cases, but information about vaccines rarely reaches them.

**Table 3:** Difference in livestock practices across ethnic groups.

**District**	**Abim**	**Amudat**	**Kotido**	**Moroto**
**Ethnicity**	**Ethur**	**Pokot**	**Jie**	**Matheniko and Tepeth**
Gender and livestock	• Generally, men own and make most decisions on livestock. • Widows still have their brothers or male in-laws take control of their livestock. • Women are less engaged in livestock activities compared to the Jie, Pokot, Matheniko and Tepeth; not even milking.	• Men are the owners of livestock. • Widows can own, control and make decisions over their livestock. • Women are engaged in and can make decisions on grazing, watering and milking. • Women are prohibited from getting near herds during menstrual periods.	• Women are engaged in and can make decisions on grazing, watering, and milking. • Widows can own, control and make decisions over their livestock.	• Generally, men own and make most decisions on livestock. • Women are engaged in and can make decisions on grazing, watering and milking.
Vaccination and information about vaccines	• More resistant and negative about vaccinations. • Information reach men more easily than women. • Most vaccination activities are done by men. • Women whose husbands are far, or widows are most likely to miss out on vaccinations.	• Are generally more responsive to vaccination and veterinary services. • Are willing and pay for veterinary services. • Information reach men more easily than women. • Widows and women whose husbands are far can drive animals for vaccination.	• Are generally more responsive to vaccination and veterinary services, • Information reach men more easily than women. • Are willing and pay for veterinary services. • Widows and women whose husbands are far can drive animals for vaccination.	• Are reluctant to pay for veterinary services. • The Tepeth live on the mountains and can't access veterinary services and vaccination sites due to geographic/topographic isolation. • Information reach men more easily than women. • Widows and women whose husbands are far can drive animals for vaccination.
Livestock raids	• Do not practice raids and have no illegal guns	• Do raids but are more defensive and retaliatory.	• The most notorious raiders in the region. • They prefer raiding other Karamojongs with guns like Matheniko, Bokora, Tepeth and Turkana of Kenya. Raiding Ethur of Abim who have no guns makes one be termed a “woman; true men need to fight and take the animals by force not with ease”. • They take raiding as sport and see it as very addictive.	• Are also notorious and sometimes they join forces with the Turkana to raid Jie and Dodoth of Kaabong.

### Social Environment Level

At this level, cultural norms regarding livestock vary greatly depending on the intersecting factors. Gender, ethnicity, age, access to education, and marital status are the main intersectional factors that determine how cultural and social norms affect an individual's ability to access PPR vaccines. Overall women are relegated to raising poultry or crops when it comes to agriculture, however, some ethnicities are more restrictive than others. For example, the Ethur ethnic group in Abim was the most restrictive of those studied. A woman from Abim said, “You will be beaten,” in response a question about their ability to sell a goat without permission of the husband. In Abim, if a man dies, his livestock does not go to his wife, but rather to his brother or other male in-laws. Other ethnic groups did not report such widespread restrictions. The Jie women in Kotido reported more engagement with livestock vaccines than women from other ethnic groups, though they were still lagging in agency over livestock compared to men. Regarding women's ownership and the ability to make decisions, among the Pokot, there was some variation between villages in whether or not a woman can be involved in raising small ruminants. Decision-making power for the sale of any livestock resides with men, but in some villages, women were able to take small ruminants for vaccination or to seek treatment from CAHWs. An additional barrier Pokot women face in accessing veterinary services is that sale of acaricides is banned in some veterinary shops in Amudat, due to high female suicide rate in contexts of intra-household violence and abuse. In a FGD with livestock keepers, one of the men said, “Women are not allowed to buy acaricides, doom, and diaconal because they can easily take these chemicals and they poison themselves out of annoyance or any misunderstanding at home.”

There are several cultural aspects that bar women from becoming CAHWs. The most common barriers are the gendered cultural norms in the household. Women's time is heavily constrained by household chores, crop production, childcare, and taking care of sick animals, all of which impede their participation in the LVVC. An important intersecting factor that hindered the ability of both men and women to become CAHW is access to education. Many CAHWs participating in FGDs couldn't write or read. Additionally, there are a myriad cultural beliefs across ethnic groups that limit women's interaction with livestock, either as owners or CAHWs. The Ethur in Abim and the Pokot in Amudat had the greatest restrictions to women becoming CAHWs. When asked about access to trainings, a woman in Abim said, “No training because we women are most time not called for training programs in livestock.” Other women said that they faced barriers due to cultural beliefs, with one woman stating, “You as a woman like us cannot enter the cattle crush to collect dung. It's a bad omen. We don't know if we are bad omens…,” while another woman said “When you are a breastfeeding mother, you are not allowed to enter the kraal because the infant animals will die.” In Amudat, one woman who owned livestock noted aloud that this was the first time she was called to a meeting regarding livestock; “This is our first time to be called for such a discussion as women, all along it was only men.” The Matheniko in Moroto and Jie in Kotido reported less restrictions when it came to women being involved in raising small ruminants or as CAHWs. Women livestock keepers share some of the same barriers with women CAHWs, such as time poverty and the cultural beliefs that livestock belong to men and women are too weak to restrain animals. Nevertheless, there are women who are highly involved in small ruminant rearing and, even without decision-making power to sell the animals, they are responsible for their health and management, sometimes going so far as procuring services from CAHWs and other veterinary service providers. Finally, another intersecting factor that hindered women becoming CAHWs is the geographic location of their home vis a vis the livestock herd, livestock communities, and the veterinary office. Due to gender based and ethnic violence, the roads for women to travel on foot to visit livestock keepers or herds are not safe. Moreover, most women do not have access to the already scarce transportation means and resources in Karamoja.

While most men (regardless of ethnic group) own and control livestock, age and physical ability are intersecting factors that affect access to vaccinations regardless of gender. Those who are old or disabled cannot drive livestock to vaccination sites, thus missing the opportunity to vaccinate their livestock. In some cases, communities reported livestock being vaccinated in the manyattas (permanent dwellings) rather than a set point to which all could bring their livestock. With this approach, vaccines come to the farmers rather than the farmers to the vaccine. This approach even though was perceived as effective for the inclusion of elders, women, and those not able to drive animals to vaccination sites, was seldom done. In all the districts studied, some participants reported that members of their communities were hesitant to vaccinate, as they thought vaccines had negative effects, such as triggering abortions, animal tails falling off, and death of animals after receiving vaccines.

### Physical Environment Level

The poor road and cold chain infrastructure of the region is a challenge for all actors in the LVVC. However, it affects livestock keepers differently depending on their ethnicity, age, physical ability, geographic location, and gender. Different ethnic groups tend to be settled in different places with different types of challenges. Livestock keepers from Moroto, both from the Matheniko and Tepeth ethnic groups, seemed to have similar factors determining their access to livestock vaccines; however, the Tepeth are additionally constrained by residing in the mountains. Being furthest away from towns and vaccination sites makes it difficult for veterinary personnel to access them (research team observed lack of roads to the Tepeth manyattas). Moreover, the geographic location makes it harder for veterinary services to mobilize and reach some Tepeth communities, as there is no cellular network in most of their settlements and infrastructure is absent. Even when mobilization is successful, Tepeth livestock keepers must travel greater distances to access vaccines. This can be discouraging as many reported that when they finally arrive to the vaccination site, the vaccines would run out, and they were not able to get their livestock vaccinated. District veterinary officials have made great efforts to ensure inclusion of the Tepeth ethnic group in vaccinations, as they were often overlooked in the past; however, logistical challenges remain a significant barrier. Some Pokot livestock keepers faced similar barriers in accessing vaccinations, as being far from town centers often resulted in exclusion or demotivation to obtain vaccines. The Pokot were the only ethnic group that mentioned participating in vaccination activities in Kenya, as their border is shared with West Pokot County in Kenya, and they move freely across the border, especially during seasonal migration.

Additionally, the intersection of geographic location with gender, age, and physical ability could further limit access to livestock vaccines among those settled further away. As previously mentioned, due to the high prevalence of gender violence, women are at higher risk when they travel long distances alone, and thus are discouraged to do so. Those who are physically impaired, or of older age, might not be able to travel long distances, which excludes them from vaccination activities. Another issue that is closely related to infrastructure and distance to veterinary services is climate, as heavy rains cause disruptions in mobility as roads become flooded. This exerts additional stress to both vaccine distributors and to livestock keepers. The security of the region, or the lack thereof, is also a significant barrier to vaccination. During data collection there were ongoing ethnic conflicts triggered by cattle raids at a small scale. These conflicts might limit the access to vaccination by ethnicity. For instance, the Jie were reluctant to accept other ethnic groups into vaccination sites during times of conflict, such as during the period of data collection.

The vaccine supply, which is the quantity of PPR vaccines available, is another factor that limits access. In all the districts studied, the lack of vaccines was highlighted as an issue; however, some were more affected than others. Men usually got information about vaccines sooner, which enabled them to reach vaccination points before women. Those with impaired physical ability would also be at a disadvantage. Another issue that is related to the vaccine supply is the lack of veterinary professionals in the region. Veterinary professionals must rely heavily on CAHWs in order to carry out vaccinations. Karamoja remains the only place in Uganda where CAHWs are allowed to operate freely and are routinely counted upon for livestock vaccinations. Although they alleviate the shortage of professionals, there are concerns among stakeholders that many CAHWs are poorly trained and that few women are involved. Livestock keepers and CAHWs expressed the need for additional CAHWs, and a need to improve the quality and recurrence of trainings.

### Policy Level

At this level, international organizations and the government of Uganda agree that eradicating livestock diseases and enabling policies that facilitate vaccination activities are of high importance. The regulatory framework has allowed the use of CAHWs in Karamoja, and it is currently working on standardizing trainings for CAHWs. However, due to the prevalence of other veterinary diseases in ruminants (small and large), vaccines for other diseases must be prioritized. Governmental economic constraints, and the lack of veterinary private sector involvement in Karamoja, leads to policies that must prioritize certain diseases, such as foot and mouth disease (FMD), or diseases for which large outbreaks have been confirmed. When vaccines for large ruminants are prioritized at the national level, all livestock keepers with small ruminants are negatively impacted, but some intersectional factors (gender, ethnicity, and marital status) serve to amplify or dampen the negative impact on individuals. Women in Karamoja do not own large ruminants and thus become excluded. This also disproportionately affects widows, as married women might still benefit from their household's cattle receiving vaccinations. Given the history of conflict and the political instability of the region, security policies are also an important factor at the Policy Level. During data collection, the escalating conflicts led to the increased presence of military personnel throughout Karamoja. As a consequence, international organizations in the region, who are actively engaged in livestock vaccination and trainings of CAHWs implemented restrictions in travel which limited CAHWs' ability to carry out activities.

### Interactions Between Different Levels

Vaccine providers rarely have enough resources to carry out vaccinations optimally, as they often lack both an adequate supply of vaccines and the logistical resources to carry out vaccinations equitably. The lack of resources leads to the prioritization of certain vaccines (e.g., FMD), preference to farmers who are logistically easier to reach, and those who have larger herds. This approach usually excludes women, poor households, and those located furthest away from where vaccinations are carried out. Some interactions occur between different levels of the SEM and are further confounded by intersectional factors exacerbating or alleviating access to PPR vaccines. For instance, the lack of mobile network coverage in certain areas (Physical Environment Level) affects ethnic groups that are settled in remote locations, such as the Tepeth. This limits the access to information about vaccination (Individual Level), and it is further exacerbated by cultural norms (Social Environment Level) that exclude women from receiving information. Another example of such interactions is vaccine prioritization. When vaccines for large ruminants are prioritized at the Policy Level, mobilization efforts to provide information about vaccination (Individual Level) focuses on men, thus creating an environment where men are more regularly engaged in vaccination activities, increasing positive attitudes toward vaccination, and limiting the interactions of women with the veterinary services. The system encompassing livestock vaccinations in Karamoja is not meant to exclude any particular group, however, the system operates with assumption that everyone has equal access, resources, knowledge, attitudes, and practices toward livestock vaccines, which is far from true. This assumption, which may be made in part due to a lack of resources and targeted interventions, leads to the exclusion of women (widows in particular), the elderly, the disabled, the ethnic minority groups, those living in geographically remote locations, and those who start to believe vaccines harm their animals. Vaccination strategies, in general, do not account for human and social dimensions surrounding the livestock keepers and as a result ignore the gender, ethnic, geographic, and societal disparities in their design and implementation of activities, thereby fundamentally limiting their reach to the most vulnerable.

## Discussion

The findings of this study highlight the importance and argue for the use of an intersectional lens in designing inclusive interventions to improve and sustain livestock health among agropastoral communities. The cultural belief that livestock management belongs to men results in mobilization and distribution of livestock vaccines almost exclusively to men. Gender has been identified as a constraint to accessing livestock vaccinations across several contexts (57–61), but very few studies have highlighted the importance of other intersectional factors beyond gender and livestock vaccines (24, 25, 32). District level stakeholders of the LVVC are trying to address some of the barriers mentioned above, however, there are barriers that are rooted in broader systemic problems. Development practitioners have identified “gender issues” as a barrier to resilience in Karamoja (62), which echoes the findings from this study, as livestock health is paramount for resilience and livelihoods in Karamoja. Our findings confirm how time poverty is highly prohibitive for women to access and actively engage in the LVVC, which could worsen given that climate change has been exacerbating time poverty constraints faced by women (63). The different factors at each level of the SEM, and how they intersect with ethnicity, gender, age, marital status, physical ability, geographic location, and access to education must be considered by stakeholders when designing and implementing interventions to increase vaccination uptake. Furthermore, underlying issues such as violence against women, result in barriers to access veterinary services (such as banning the sale of acaricides in certain parts of Amudat), but more importantly, it also affects the physical and mental wellbeing and self-worth of women. Rates of alcohol consumption among women have been in an upward trend, which some suggest might be due to food insecurity and worsening of general wellbeing of the household (64). Addressing the root causes of gender violence in Karamoja must become a policy priority, as these issues have deep ramifications that affect the health and livelihoods of all.

In Uganda, gender equality is defined as joint household decision-making but for the most part, men still make the decisions and consensus is the wives doing what the husbands say (65). As evidence of the prevalence of gender and intersectional issues in the Karamoja subregion, few to no women apply or obtain positions related to animal husbandry or veterinary services at the district level, both because there is a lack of qualified applicants, and the still deeply engrained cultural belief that veterinary medicine is better suited for men. This is deeply rooted in the patriarchal beliefs of the Karamojong, which, even after the disarmament and the extensive work by NGOs to educate about women's empowerment, persist in communities across Karamoja (66). Furthermore, education levels in Karamoja are low, as most children do not go to primary school. Over 70% of the population aged 10 or more has never been to school, and most of them are women (15). Low levels of education also thwart vaccination efforts, as lack of knowledge by livestock keepers about vaccines and vaccination benefits makes their mobilization efforts harder. The role that women play in livestock production and animal health goes unrecognized in Karamoja and, as such, their participation in vaccinations is low. Only a few women are known to be involved in livestock vaccinations, and, for the most part, they are those carrying water to the vaccination site. This marginalization has made them less involved overall, which leads to even less knowledge about vaccines and veterinary services. It is unclear what proportion of women have control or decision-making power over animals, as ownership and management of livestock in Karamoja is very nuanced (67). However, the fact remains that some women, such as widows, or those who acquire livestock through NGO or government projects, suddenly become part of the livestock value chain. These women are at a great disadvantage compared to their male counterparts, as they have had less exposure to veterinary services, but more importantly remain neglected by veterinary services. Animals owned and managed by women remain low priority to veterinary services constrained with limited resources and vaccine supply, as veterinary services tend to prioritize large ruminants. Women rarely have the agency to sell livestock without permission from their husbands, even when the livestock belong to the women. All these barriers contribute to women being almost invisible to veterinary services and/or vaccination programs. Those providing vaccines are already stretched and have little incentive to mobilize women for vaccination as they have less animals, less assets, and face greater barriers to drive them to the vaccination site. Even though evidence from our results showed that vaccine providers do not discriminate against women when they bring animals to vaccination, little effort is done to ensure information about livestock vaccines reaches women, or that the vaccination process is one that enables women to participate. It is perceived that involving women in vaccinations in Karamoja is a high-cost/low-benefit scenario. Most importantly, the degree of participation in the LVVC is not only influenced by the factors outlined in the SEM and gender alone, but by the intersection of gender and ethnicity, age, marital status, physical ability, geographic location, and access to education. These intersections are important when undertaking vaccination efforts. Those designing and implementing vaccinations should take into consideration these nuances to tailor their interventions as a one-size-fits-all approach for the Karamoja subregion would not be effective. Not all livestock keepers in Karamoja share the same barriers to vaccination, men and women have different barriers, but within those two broad categories there are many more distinctions that need to be accounted for to make vaccination activities equitable, fair, and inclusive.

Regarding women's participation as CAHWs, they remain constrained by cultural beliefs, gender-based violence, and affected by time poverty. This is problematic, as women CAHWs are a key in communicating information to female livestock keepers, which is why some women livestock keepers in FGDs expressed their need for more female CAHWs within their communities. A parallel study in Senegal found cultural beliefs and time poverty barriers for livestock keepers when accessing the LVVC, with women and those living in the most remote areas having overall less access to vaccines (25). The neglect that some groups face when it comes to livestock vaccines can result in pockets of unvaccinated livestock where diseases, such as PPR, can become entrenched and never fully be eliminated. While the percentage of small ruminants owned by women and other vulnerable groups is small, they could remain a reservoir for PPR and other diseases if not vaccinated, hindering the global eradication efforts. It is important that those in charge of mobilization of livestock keepers start reaching out to those who have been historically marginalized and neglected when it comes to any veterinary service and information. The systematic exclusion of women and other vulnerable groups has led to a lack of knowledge of veterinary practices. The continued exclusion from routine veterinary services could increase vulnerabilities by limiting the access and information about vaccines and other livestock health related services.

### Limitations of the Study

In some cases, it was not possible to have a women's only CAHW FGDs due to how few there were in certain districts at the time of data collection. Mixed FGDs, or individual interviews were done in place when not enough women could be mobilized, trying to create a safe and comfortable ambience for women to talk freely, however it remains uncertain if these were achieved. Another limitation was that some livestock keepers had rarely received vaccinations for small ruminants, and their engagement with veterinary services was very limited, or only limited mostly to large ruminants. Furthermore, local languages had different names for diseases, and in some cases diseases such as PPR were named based on the animal's symptoms, which are common symptoms with other diseases. This required an introductory session of participatory epidemiology which usually extended the duration of the FGDs. There could have been also meeting fatigue, especially among male participants, given the increasing number of interventions in the region might reduce the willingness of inhabitants to constantly participate. The increasing amount of aid and interventions regarding livestock could also introduce some level of response bias.

## Conclusions

Livestock keepers from Karamoja are an ethnically diverse and complex group of people with different socio-cultural beliefs and gender norms. Livestock vaccination trainings and campaigns rarely include intersectional considerations. Furthermore, women and other vulnerable groups rarely participate in trainings or other extension activities related to livestock. There are significant gender and ethnic differences in Karamoja, however, these differences are seldomly considered in vaccination and other veterinary activities. Overall, a set of tailored interventions regarding animal health, including livestock vaccines, is recommended, as cultural beliefs and knowledge is highly variable across the region. Women in general face other issues of more immediate need than being left out of livestock vaccinations. Most projects or interventions in the region aim to address issues such as low access to education, gender-based violence, HIV, food insecurity, and income generating activities. However, it is important to work in parallel on sustainable long-term solutions for issues regarding livestock management, such as inclusion of women and other vulnerable groups in livestock related activities, as this is a clear path to increase resilience and livelihood security.

## Data Availability Statement

The datasets presented in this article are not readily available because Data are subject to embargo until 6 months after the end of the project (which is March 2023) and will be shared publicly afterwards. Data are stored in GatorCloud storage space within One Drive@UF in observation of University of Florida's Research Data Management Policies. Authors can make data available for researchers who meet the criteria for access to confidential data. Regarding data access, we want to clarify why the qualitative data from the interview and focus group discussions reflected in this paper are available upon request. Participants have been told that all information will be kept confidential and only used for the purposes of this research. Moreover, meta-analysis of these data across three countries is anticipated at midline and endline (2023) of this study, therefore investigators request an embargo on data until 6 months after project completion to allow for final treatment and analysis of data by the research team. Data requests may be sent to the Principal Investigator of the project, Sandra Russo. Requests to access the datasets should be directed to Sandra Russo, srusso@ufl.edu.

## Ethics Statement

The studies involving human participants were reviewed and approved by University of Florida's Institutional Review Board, Higher Degrees Research Committee at the Makerere University, and Uganda National Council for Science and Technology. The participants provided their written informed consent to participate in this study.

## Author Contributions

NL, DA, SM, and SR contributed to the design of the data collection instruments and drafting and review of the manuscript. NL and DA supervised and participated in the data collection and led the data analysis. All authors contributed to the article and approved the submitted version.

## Funding

The research was funded by the Livestock Vaccine Innovation Fund under a project titled Advancing women's participation in livestock vaccine value chains in Nepal, Senegal and Uganda Grant No. 109062-001. The Livestock Vaccine Innovation Fund is supported by the Bill & Melinda Gates Foundation (BMGF), Global Affairs Canada (GAC), and Canada's International Development Research Center (IDRC). The views expressed herein do not necessarily represent those of IDRC or its Board of Governors.

## Conflict of Interest

The authors declare that the research was conducted in the absence of any commercial or financial relationships that could be construed as a potential conflict of interest.

## Publisher's Note

All claims expressed in this article are solely those of the authors and do not necessarily represent those of their affiliated organizations, or those of the publisher, the editors and the reviewers. Any product that may be evaluated in this article, or claim that may be made by its manufacturer, is not guaranteed or endorsed by the publisher.

## Abbreviations

CAHW: Community Animal Health Worker
FGD: Focus Group Discussion
II: Individual Interview
KII: Key Informant Interview
LVVC: Livestock Vaccine Value Chain
PPR: Peste des petites ruminants
SEM: Socio-ecological model.
