# Supplementary material for: Who Has Access to Livestock Vaccines? Using the Social-Ecological Model and Intersectionality Frameworks to Identify the Social Barriers to Peste des Petits Ruminants Vaccines in Karamoja, Uganda
Source: Front Vet Sci. 2022 Feb 28;9:831752. doi: 10.3389/fvets.2022.831752 (PMC8918586; doi:10.3389/fvets.2022.831752)
Supplement: Supplementary file 1 [file Presentation_1.PDF]

Appendix 1. Interview Protocols

Gender and Livestock Vaccines Value Chain Analysis in Nepal, Senegal, and Uganda
Community animal health worker focus group discussion
University of Florida

Background information:
Date_____________ and Time: __________________
Location (village, district, province, country): _________________________________
GPS Coordinates: LAT: ___________ LONG: ___________
Total number of participants at start: Women _____ Men ______
Facilitator's name: _______________________
Translator's Name: __________________
Note taker's name: ________________________

Icebreaker activity:
Before we begin this discussion, I would like to better understand your community. Can you please, using markers, paper, and words, describe *your community?*

- *Probes: grazing areas, water points, veterinary services, health services, schools, paved and unpaved roads, markets, type of livestock animals common, prevalent livestock diseases if known, number of livestock owners, ask the number of female and male livestock owners.*

Focus group discussion topics

1. Would you please describe how livestock vaccinations take place here? *Probe for:*

- *What vaccines*
- *For sale or free*
- *By whom (sale/gift)*
- *Provider*
- *Demand (by gender, ethnicity, or other)*
- *Variability in delivery system*

2. I'd like to understand more about your inventory, supply, and demand. Can you please describe a typical week, in terms of how much vaccine you might keep, dispense, sell; what records you keep, and so on. *Probe for:*

- *Frequency of restocking*
- *Record keeping*
- *Number of vaccinations daily/weekly*
- *Storage of vaccine (place and mechanism)*
- *Livestock keeper practices of vaccination – how typical? What portion of herd?*

3. Can you please describe the constraints you've experienced in providing vaccination services? *Probe for:*

- *Distance*
- *Infrastructure*
- *Refrigeration*
- *Cultural believes or norms*
- *Compensation*
- *Time/scheduling*

4. What are the advantages and disadvantages of having vaccines available within the community, by community animal health workers, vet shops, agro-vets? *Probe for:*
- *Location*
- *Geographical coverage*
- *Seasonality*
- *Benefits*
- *Who gets excluded?*

5. Now let's talk about people who buys or uses vaccinations. Help me understand how people choose to vaccinate or not vaccinate their animals. *Probe for:*
- *Why? When? How?*
- *Which animals*
- *Motivation (outbreaks, availability, seasonality etc.)*
- *Community animal health worker support in decision*

6. In your community, which households or individuals face the greatest barriers to accessing livestock vaccines? Why? *Probe for:*
- *Sex*
- *Socio-economic status*
- *Location*
- *Ethnicity*
- *Caste*
- *Religion*
- *Cultural belief*

7. What is it like to be a community animal health worker in this area? What do you like about being a community animal health worker? What parts are difficult? *Probe for:*
- *Gender (proportion as well as experience)*
- *Experiences of being a community animal health worker*

8. Some people are thinking of having centers where people can access both veterinary and human health services, such as vaccines for children and animals. What do you think about this idea? *Probe for:*
- *Would it work here? Why/Why not?*

9. Are there any other issues with vaccines we have not talked about?

Gender and Livestock Vaccines Value Chain Analysis in Nepal, Senegal, and Uganda
Livestock keepers' focus group discussion
University of Florida

Background information:
Date_____________ Time ________________
Location (village, district, province, country): __________________________________
GPS Coordinates: LAT: ___________ LONG:___________
Total number of participants at start: Women _____ Men ______
Facilitator's name: _______________________
Translator's Name: __________________
Note taker's name: ________________________

Icebreaker activity:
Before we begin this discussion, I would like to better understand your community. Can you please, using markers, paper, and words, describe *your community?*

- *Probes: grazing areas, water points, veterinary services, health services, schools, paved and unpaved roads, markets, type of livestock animals common, prevalent livestock diseases if known, number of livestock owners, ask the number of female vs male livestock owners.*

Focus group discussion topics

1. Would you please describe how livestock vaccinations take place here? *Probe for:*

- *What vaccines*
- *For sale or free*
- *By whom (sale/gift)*
- *Provider (Preference by livestock keepers?)*
- *Demand (by gender, ethnicity, or other)*
- *Variability in delivery system*

10. In thinking about your experiences with veterinary vaccination services over the past year, can you please describe your engagement. *Probe for:*

- *Frequency of use*
- *By whom*
- *Herd coverage*
- *Species type/vaccine type*

11. Who provides veterinary services and what are your constraints in accessing them? *Probe for:*

- *Price/cost*
- *Distance*
- *Infrastructure*
- *Refrigeration*
- *Cultural believes or norms*
- *Gender/caste*
- *Seasonality*
- *Time*

12. What are the advantages and disadvantages of having vaccines available within the community, by community animal health workers, agrovets, or vetshops? *Probe for:*

- *Location*

- *Geographical coverage*
- *Seasonality*
- *Benefits*
- *Who gets excluded?*

13. We spoke briefly about constraints to veterinary services, and I'd like to better understand what motivates people to buy or use vaccinations. What drives people to choose to vaccinate or not vaccinate their animals? *Probe for:*

- *Seasonality*
- *Outbreaks*
- *Availability*
- *Advice (elders, extension, community animal health workers' guidance)*
- *Cost/perceived risk*

14. In your community, which households or individuals face the greatest barrier to access livestock vaccines?

- *Sex/gender/female headed housholds*
- *Socio-economic status*
- *Location*
- *Ethnicity*
- *Caste*
- *Religion*
- *Cultural belief/Stigma*

15. What is your experience working with community animal health workers/para-veterinarians/agro-vets? *Probe each if available in the community.*

16. Some people are thinking of having centers where people can access both veterinary and human health services, such as vaccines for children and animals. What do you think about this idea? *Probe for:*

- *What would be the benefit of such co-location?*
- *What would be the problems?*

17. Are there any other issues with livestock vaccines we have not talked about?

18. Now, I have a few more questions to solicit your input in understanding how your community defines an empowered woman and disempowered woman. *Probe for:*
    - How do you define being empowered?
    - How do you define being disempowered?
    - What does it mean to your personally?
    - *Probe further if necessary:* Who would you say in your community is empowered? How is disempowered?

19. Can you explain what types of livelihoods are common in your community? How do you define each?

20. Who is in charge of livestock in your community or household? What does the word "in charge" mean to you?

Gender and Livestock Vaccines Value Chain Analysis in Nepal, Senegal, and Uganda
Individual questionnaire for community actors in the value chain
University of Florida

Background information:
Date_____________ Time ________________
Location (village, district, province, country): _________________________________
GPS Coordinates: LAT: ___________ LONG: _____________
Respondent's Gender: Woman _____ Man ______
Translator's Name: __________________
Note taker's name __________________
Interviewer's name _________________

Module A: *[Ask all]*

1. What is your role in caring for livestock health?
   Response:
   - Retailer of veterinary products
   - Farmer/livestock keeper
   - Village/Community Animal Health Worker
   - Other: [*Specify*] ________________________

2. How long have you been in this line of work?

3. What reasons led you to become, and *how* did you become, a _____________________ [*fill in based on answer for question 1*]?

4. What type of training have you received related to your role [*whether formal or informal*]? If yes, please describe who delivered it, what the training was about and approximate date of last training.

5. Have you received any business training? If yes, please describe who delivered it, what the training was about and approximate date of last training.

6. What are the vaccines you sell/use in your work the most? List the top five.

7. Where do you get these vaccines? [*Check the source of all top five if provided for question 6*.]
   a. Are they always available? Why or Why not?

8. [*If not mentioned in questions 6 and 7, ask*] Where do you get vaccines for PPR and New Castle diseases?
   a. Are they always available? Why or Why not?

9. What do you do with the livestock vaccine once you have it? [*Check all that apply*]
   Response:
   - Vaccinate livestock
   - Store
   - Re-package for use
   - Sell
   - Other: [*Specify*] ________________________

Module B: [*If interviewee is a community animal health worker or agro-vet owner*]

1. What other services related to livestock do you provide, besides vaccines [*or vaccination services*]?

2. Did you participate in livestock vaccination campaigns in the past year? What campaign (s)? What vaccines? How many animals? Which members of the household typically come to you to get vaccines? Men? Women?

3. Is it usually the same person in the household who decides about vaccinating animals? Do women approach you for vaccines [*or vaccination services*], and if so, which vaccines?

4. How easy is it for community members to access your services? Please explain.
(For Senegal: ask Questions 5 and 6; other countries: skip)

5. Do you also sell [*provide vaccination services for*] human vaccines/medicines?

6. Do pharmacists also sell veterinary medicines and/or vaccines?

7. In your position, do you work with women?
Response:
    - No
    - Yes

   If yes, give the approximate number of women______
   If not, give approximate number of men _________

8. Are there community animal health workers or agro-vets in your area? How many? How many are women? If agro-vet shops, then women-owned?

Module C: Demographic Questions *[Ask all]*

1) What is your age in years? *(Circle correct response)*

   Less than 20 20-2 25-29 30-34 35-39 40-44 45-49 50-54 More than 55 Don't know

2) What is your marital status? *(Circle correct response)*
   Single Widowed Married customarily Married civil Married in polygamous marriage Divorced

   - *[If married, see below A for men and B for women]*
     A. How many wives do you have?
     B. Are you the only wife? If not, how many wives does your husband have?

3) Please specify your ethnicity: ___________

4) Please specify your religious beliefs: _________

5) Are you the head of household? *(Household is defined as the set of people who live in the same house or compound)*

6) What is your level of education? *(Circle correct response)*
   a. Didn't attend school
   b. Attended primary school (1-6)
   c. Completed primary school
   d. Attended secondary school
   e. Completed secondary school
   f. Higher than secondary
   g. Did you attend a vocational school or other informal education? ___ YES ___ NO

7) What is the highest level of education attained by any member of your household?

8) Including yourself, how many people live in your household?

9) How many adults (+18) live in the household?

10) How many children (under 18) live in the household?

11) What is the main source of income for the household? *(Circle correct response)*
Cash Crops Food Crops Livestock Raising Fishing/Aquaculture Non-farm business/trade Wages/salaries

12) What is the secondary source of income for the household? *(Circle correct response)*
Cash Crops Food Crops Livestock Raising Fishing/Aquaculture Non-farm business/trade Wages/salaries

Gender and Livestock Vaccines Value Chain Analysis in Nepal, Senegal, and Uganda
Key informant interview questions
University of Florida

Background information:
Date_____________ Time _____________
Location (village, district, province, country): __________________________________
GPS Coordinates: LAT: ___________ LONG: ___________
Respondents Gender: _____________
Translators Name: ___________________
Interviewer’s name: ___________________
Notetaker’s name:___________________

Module A: Livestock vaccine value chain

1) Can you describe the organization or project you work in?

2) Can you describe, in few words, your role in your organization or project?

3) How long have you been working in this position or similar?

4) Geographically, where do you mostly work?

5) What livestock vaccines are distributed in your area?

6) What vaccines have higher priority? Why? Who decides this?

7) Are there regions or districts that receive more vaccines than others? Who decides the amount that goes to each region or district?

8) Describe the livestock vaccine distribution system in your country or area (*probe from production point, and entry point into the country all the way to delivery to farmers)*.
   a. Who are the actors involved in the distribution system?
   b. What steps are involved?
   c. Where do you and your organization fit in the system?
   d. When are vaccines distributed? *[probe: an outbreak, regular vaccination scheme, seasonality, particular funding from a donor, etc.]*

9) What do you think are the main problems or bottlenecks in this system? *[Select all that apply and explain]*
   - ☐ Availability
   - ☐ Quality
   - ☐ Cost
   - ☐ Supply delays
   - ☐ Storage or refrigeration
   - ☐ Transport
   - ☐ Government certification or registration process for vaccines
   - ☐ Availability of vaccinators
   - ☐ Others, *please specify:* ___________________

10) Do these constraints affect women and men in the system differently? Explain. *[This is about the actors in the distribution system]*

11) Of these systems wide constraints, which ones affect your work the most? *[Notes: list and explain the three most important constraints for each vaccine*

12) What are the main constraints farmers face getting vaccines? *[Notes: explain as many as possible and rank top three most important constraints]*

13) What type of households face greater difficulties to access vaccines? Does access to vaccines depend on gender, socio-economic status, location, ethnicity, religion, livelihood? *[probe on Intersectionality aspects characteristic to the area]*

14) Is there a mandate from the government or donor that specifies certain sub-groups receiving livestock vaccines? Or is there a special effort within your organization to increase the uptake of livestock vaccines among specific types of livestock keepers, e.g., female livestock owners? Why or Why not?

15) We would like to understand how you collaborate with other organizations. Do you work in cooperation with: *[Explain and elaborate]*
- ☐ Government agencies
- ☐ Non-government organizations (*probe for local, national, international*)
- ☐ Private sector
- ☐ Farmer organization

Module B: Empowerment domains for value chain actors with regard to empowerment in their organizations

16. One of the focuses of our research is to understand the barriers and opportunities for women's participation in the livestock vaccine value chains. In your opinion, does your organization or project do any work in this area? *Probe for:*
- If no, do you think that they should be working in this area?
- If yes, how are they performing in this area?

17. To what extent does the leadership in your organization or project provides strategic direction to mainstream gender to *[name activities]?*

18. How many women work in your organization or project? Why or why not?

19. Would you like to see more women in your organization or project? Why or why not?

20. Is there anything else you would like to share with me today?
